# Requirements to Practice Dentistry in Foreign Countries

**Published:** 1897-01

**Authors:** 


### Editorial, Etc.

### REQUIREMENTS TO PRACTICE DENTISTRY IN FOREIGN COUNTRIES.

The bureau of statistics of the State Department has gathered recently a great deal of valuable and interesting information in regard to the practice of dentistry in foreign countries, and especially in relation to the qualifications necessary for an American dentist to practice abroad. The data was obtained through the diplomatic officers at the European capitals.

In a letter to Mr. Bayard, our ambassador to Great Britain, Lord Salisbury wrote, under date of August 1, that "the Genenal Medical Council had decided to recognize only such certificates as were evidence of a course of study and examination in arts and in dental surgery not inferior to those of British dental practitioners."

He added that "as the result of an inquiry held by them in 1879 into the sufficiency of the degree granted by the various American dental-licensing bodies who applied for recognition, the University of Harvard and University of Michigan were recognized as bodies granting 'qualifications' which entitled the holders to registration in the

United Kingdom, and no other application was granted. But after investigation in 1893 by the education committee of the county it was found that the curricula of these two bodies were not considered adequate to insure the possession of the necessary qualifications, and the council thereupon resolved that the recognition of the dental degrees of the two universities above mentioned should be suspended until further notice. Consequently no foreign dental degrees are at present recognized."

In Switzerland no foreign diploma is recognized. All foreigners desiring to practice dentistry must submit to a thorough practical examination, conducted in the French German or Italian language. The practical effect of these regulations is the exclusion of all, or nearly all, American dentists, as a residence of several years in Switzerland or a regular course in one of their universities would be necessary to qualify them to successfully pass an examination. In answer to the remonstrance raised by American dentists there against these regulations, the response is made that identically the same requirements are exacted of native practitioners.

Swedish citizenship is required for every one wishing to practice a profession in Sweden, but there is nothing to prevent surgeon dentists, wishing to practice under the rules imposed upon Swedish dentists, addressing a supplication to the King stating their intention of becoming Swedish citizens. Applications must always be accompanied by certificates and the applicants must pass the regular examination. There have been many such applications presented, but very few instances of permission having been granted.

In Spain foreigners who desire to practice dentistry must present the degree acquired in a foreign public institution and pay 200 escudos on obtaining the authorization, which shall be given on receipt of the certified degrees. The foreign degree must be one that is recognized as valid in Spain. Application must be made on stamped paper, addressed to his excellency the minister of public works, accompanied by the original degree and the translation of it into Spanish, made by the interpreter at the department of foreign affairs.

In Portugal a rigorous examination in the Portuguese language is imperative for all persons who desire to practice medicine or surgery in any of its branches. Dentists are examined closely as to the anatomy of the head. If they pass successfully they are entitled to practice upon the payment of 60 dollars.

An examination is required in Russia, the candidates having shown satisfactorily that they have passed through the classes of science relating to the degree or title they possess. The examinations include operations on dead bodies and living beings.

In order to practice the profession of dentistry in Mexico it is not necessary to previously undergo an examination, it being sufficient to have the proper diploma issued by a foreign dental college and the certificate, duly authenticated by the competent authority, in consideration of which that college has the legal power to issue such diplomas. Neither do the Mexican laws require the interested party to pass an examination in the Spanish language.

In the Netherlands a candidate must have a diploma before he can appear before the examining board. The foreign certificates or diplomas thus recognized are the Belgian, German, British, French, Austrian and Swiss medical diplomas; and the Belgian, German, British, French and Swiss dental diplomas, in addition to those issued in the Dutch Indies, Surinam and Curacao.

In Italy, according to a letter from our Secretary of Embassy at Rome, there is no regular school of dentistry and some little informality in practice has existed, at least locally in Rome, in permitting foreigners to follow our profession, and on presentation before the proper officials of satisfactory credentials the applicants have been told they might practice among the people of their own nationality. But lately there has been begun an agitation against the granting of even that limited privilege.

All persons desiring to obtain permission to practice dentistry in Greece must submit an official certificate from a recognized dental school or from a recognized dentist, certifying that they have been instructed and are proficient in dentistry. Their examination shall be conducted either in the Greek language or in a foreign language. Foreign diplomas are authenticated on payment of 400 drachmas (about $46) for the stamped paper required. Without previous authentication of their diplomas, applicants from foreign countries are not allowed to present themselves for examination.

No official permit is necessary to practice dentistry in Germany, but for the title of doctor of dentistry an examination is required.

No one can exercise the profession of dentist in France if he is not provided with a French diploma of doctor of medicine or of surgeon dentist, issued by a superior medical institution of the State, after a certain course of studies fixed by the government and followed by public examination.

Foreign dentists are practically excluded from practicing in Austria. Every medical doctor or dentist must have passed the regular examinations of the various Austrian State preparatory schools university and medical college. No foreign diplomas of any kind are accepted as evidences of fitness in either profession.

In Belgium any foreign candidate who has acquired the right to practice his profession in his own country may be admitted to an examination, and if he passes he may practice his profession without further preliminaries.

Denmark requires a license, which cannot be obtained without passing an examination.

In order to practice dentistry in Hawaii a creditable examination before a board of dental examiners is required, but a certificate of qualification will be given to any person who presents a diploma from a reputable dental college.

